# 4D Multi-Modality Tissue Segmentation of Serial Infant Images

**DOI:** 10.1371/journal.pone.0044596

**Published:** 2012-09-25

**Authors:** Li Wang, Feng Shi, Pew-Thian Yap, John H. Gilmore, Weili Lin, Dinggang Shen

**Affiliations:** 1 Department of Radiology and BRIC, University of North Carolina at Chapel Hill, Chapel Hill, North Carolina, United States of America; 2 Department of Psychiatry, University of North Carolina at Chapel Hill, Chapel Hill, North Carolina, United States of America; Institute of Psychology, Chinese Academy of Sciences, China

## Abstract

Accurate and consistent segmentation of infant brain MR images plays an important role in quantifying patterns of early brain development, especially in longitudinal studies. However, due to rapid maturation and myelination of brain tissues in the first year of life, the intensity contrast of gray and white matter undergoes dramatic changes. In fact, the contrast inverse around 6–8 months of age, when the white and gray matter tissues are isointense and hence exhibit the lowest contrast, posing significant challenges for segmentation algorithms. In this paper, we propose a longitudinally guided level set method to segment serial infant brain MR images acquired from 2 weeks up to 1.5 years of age, including the isointense images. At each single-time-point, the proposed method makes optimal use of T1, T2 and the diffusion-weighted images for complimentary tissue distribution information to address the difficulty caused by the low contrast. Moreover, longitudinally consistent term, which constrains the distance across the serial images within a biologically reasonable range, is employed to obtain temporally consistent segmentation results. Application of our method on 28 longitudinal infant subjects, each with 5 longitudinal scans, shows that the automated segmentations from the proposed method match the manual ground-truth with much higher Dice Ratios than other single-modality, single-time-point based methods and the longitudinal but voxel-wise based methods. The software of the proposed method is publicly available in NITRC (http://www.nitrc.org/projects/ibeat).

## Introduction

The first year of life is the most dynamic phase of postnatal brain development. The brain undergoes rapid tissue growth and experiences development of a wide range of cognitive and motor functions. Accurate tissue segmentation of infant brains in the first year of life has important implications for studying normal brain development, as well as for diagnose and treatment of neurodevelepmental disorders such as attention-deficit/hyperactivity disorder (ADHD), and autism. Current methods are able to segment neonates (less than 3 months) and infants (over 1-year-old) with great success [1]–[8] (a summary can be found in [9]). In general, most existing neonatal segmentation methods were proposed for single time-point image (less than 3 months). Few algorithms take advantage of the increasing availability of longitudinal MR images, which provides important information on the evolution of brain structures. Moreover, most existing methods rely on the T2 modality for the neonates less than 3 months old [4], [5], [8] and T1 modality for the infants over 1-year-old [10], [11], which demonstrates good contrast between white matter (WM) and gray matter (GM). However, at the middle of the first year (around 6–8 months of age), T2 and T1 modalities have lowest contrast where the WM and GM exhibit almost the same intensity level, which poses a significantly more challenging problem [12].

**Figure 1:**
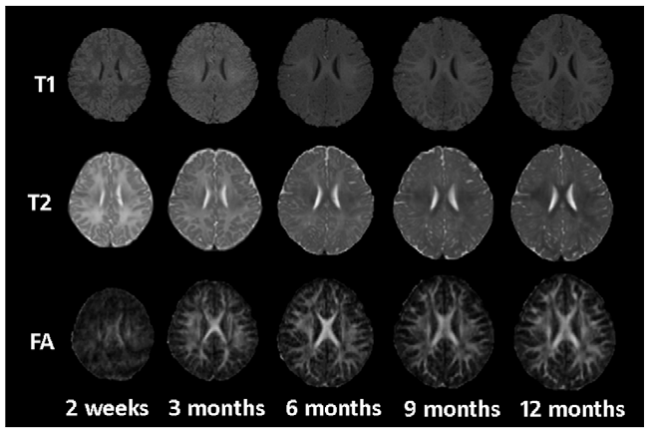
T1, T2 and FA images of an infant scanned at 2 weeks, 3, 6, 9 and 12 months.

During the brain growth in the first year, the intensity contrast between WM and GM dramatically reverses owing to maturation and myelination. There are three distinct WM/GM contrast patterns that can be observed in images of normal development infants (in chronological order) [13]: infantile (birth), isointense, and adult-like (10 months onward). As an illustration, we show in Fig. 1 a series of longitudinal MR images for an infant scanned every 3 months, starting from the second week. From the T1 images, it can be seen that the intensity of WM is initially lower than that of GM, but becomes gradually brighter, resulting in a contrast pattern that resembles adults. The opposite trend can be observed for T2 images. At around 6–8 months of age, the WM and GM exhibit almost the same intensity level (see the third column of Fig. 1), resulting in the lowest WM/GM contrast and hence great difficulties for segmentation. Few studies have addressed the tissue segmentation problem of the isointense infant images, hindered by the insufficient contrast of the respective T1/T2 images [12].

The fractional anisotropy (FA) images from diffusion tensor imaging (DTI) (last row of Fig. 1) provide rich information of major fiber bundles [14], especially in the subcortical regions where GM and WM are hardly distinguishable in the T1/T2 images. Moreover, the WM structure remains very consistent throughout all time points, proving partly the notion that the majority of the fibers exist at birth. In this work, we employ complementary information from multiple modalities by using T1, T2 and FA images to deal with the problem of insufficient tissue contrast. Information from these images are fed into a longitudinally guided level-set-based framework for consistent segmentation of the serial infant images. The main idea of level set method is to embed a front or interface of interest as the zero level set of a higher dimensional function [15]. To introduce temporally consistent segmentation results, we enforce a longitudinal constraint term that is in accordance to the fact that global brain structures of the same full-term infant are closely preserved at different developmental stages [16]. The constraint keeps the distance between the tissue boundaries of the serial images within a biologically reasonable range. This approach was tested on 28 infants with longitudinal acquired images.

**Figure 2:**
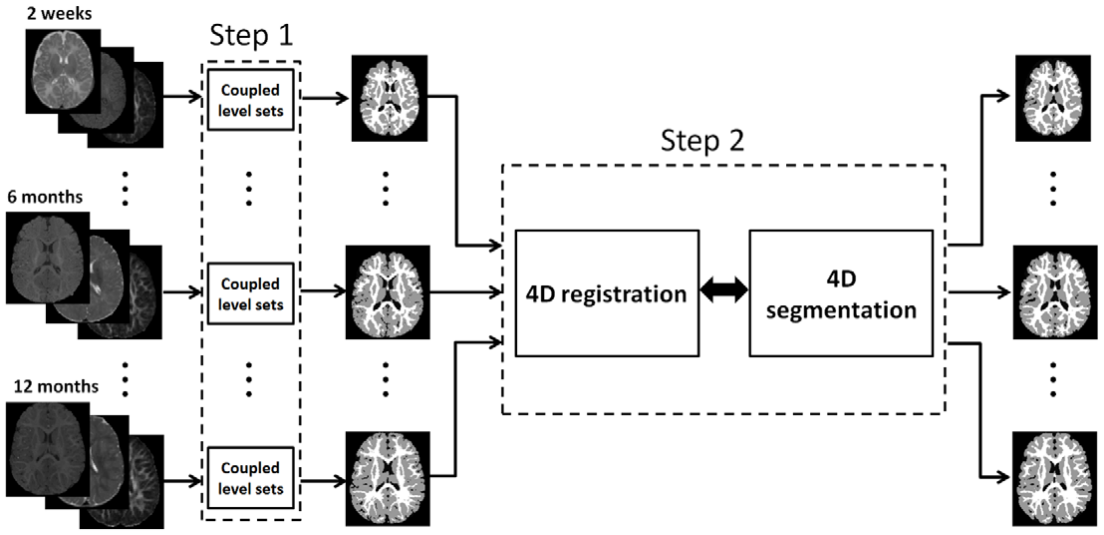
The proposed framework for segmentation of serial infant brain MR images.

This paper is an extension of our previous work [17], which focuses on the image segmentation of neonates (e.g., 2 weeks) by using subject-specific guidance from follow-up images (1-year-old or 2-year-old). In the current work, the segmentation of the images at each time-point will be influenced by the neighboring time-points. Both the early time-point (e.g., neonate) and late time-point (e.g., 1-year-old) images provide guidance for the segmentation of middle year (e.g., 6-month-old) images. In addition, to alleviate the problems associated with contrast especially for images at isointense stage, we propose to integrate the information from multiple modalities (T1, T2 and DTI). Note especially that the FA images from DTI provide rich information of major fiber bundles, which can be used to improve the segmentation accuracy.

**Figure 3:**
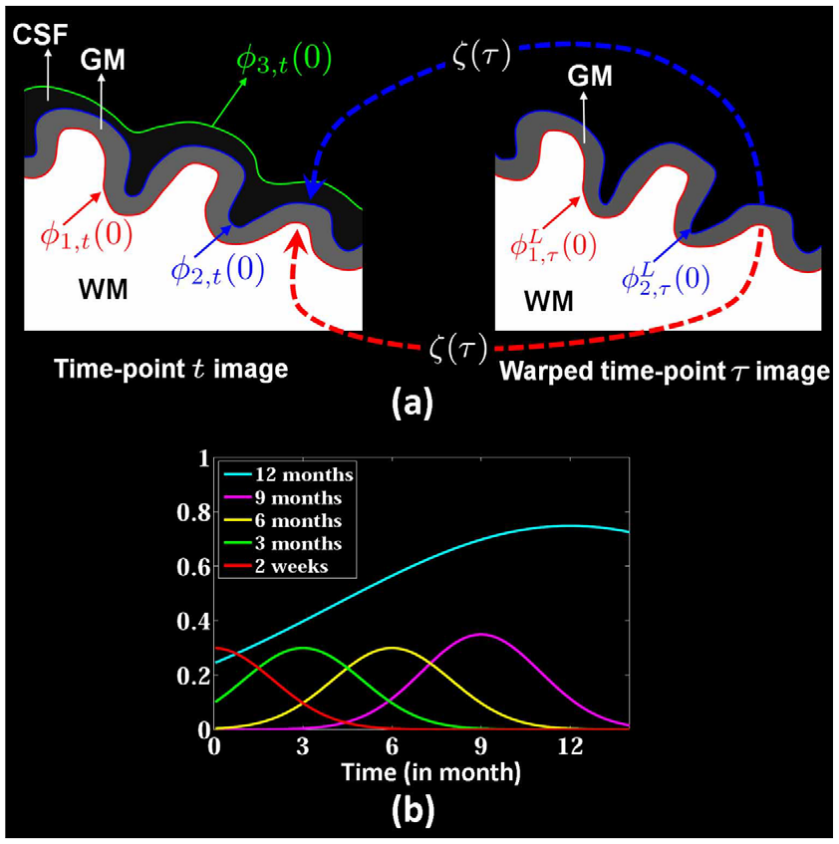
(a) Longitudinal guided level-sets segmentation. The evolutions of 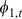 and 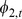 are not only influenced by the information from the current image but is also adaptively constrained by longitudinal information from the image at another time-point 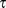, weighted by 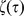, where 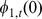, 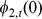 and 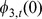 are the interfaces between WM/GM, GM/CSF, and CSF/Background, respectively. (b) The weight parameters 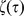 for the image at each time-point (the last time point is 12 months).

## Materials and Methods

### Ethics Statement

Subjects used in this paper were part of a large study of early brain development in normal children [18]. This study was approved by the ethics committee of University of North Carolina (UNC) School of Medicine. The parents were recruited during the second trimester of pregnancy from the UNC hospitals and written informed consent forms were obtained from all the parents. The presence of abnormalities on fetal ultrasound, or major medical or psychotic illness in the mother, was taken as exclusion criteria. The infants were scanned without being sedated, fed before scanning, swaddled, fitted with ear protection, and had their heads secured in a vacuum-fixation device. A physician or nurse was present during each scan; a pulse oximeter was used to monitor heart rate and oxygen saturation.

**Figure 4:**
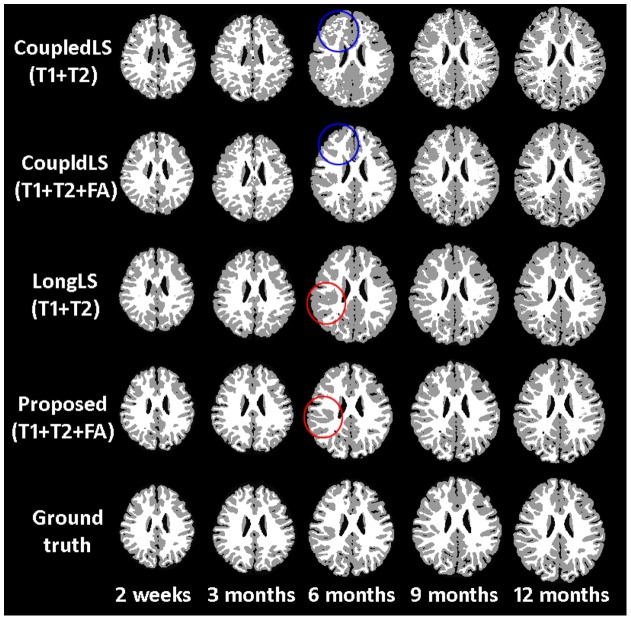
From top to bottom show the segmentation results of *CoupledLS(T1+T2)* [**22**], *CoupledLS(T1+T2+FA)* [**22**], *LongLS(T1+T2)* [**17**], the proposed method and manual ground truth. The original images are shown in Fig. 1. The blue circles mark the obvious difference between the results given by *CoupledLS(T1+T2)* and *CoupledLS(T1+T2+FA)*, while the red circles mark the obvious difference between the results given by *LongLS(T1+T2)* and *Proposed(T1+T2+FA)*.

**Figure 5:**
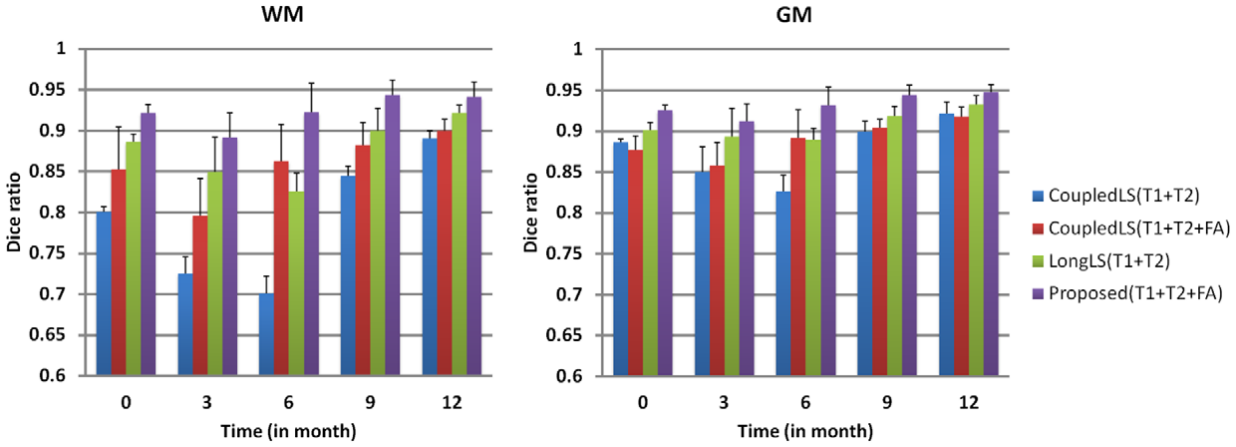
The average Dice ratios of different methods on 28 subjects. The proposed method achieves significant (p
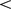
0.01) higher Dice ratio than other methods.

### MRI Acquisition

Images were acquired on a Siemens head-only 3T scanner (Allegra, Siemens Medical System, Erlangen, Germany) with a circular polarized head coil. Each subject was scanned at 5 time points: 2 weeks, 3, 6, 9, and 12 months (or older than 12 months). T1 images were acquired using a 3T head-only MR scanner, with 144 sagittal slices at resolution of 1
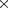
1
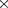
1mm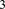, TR/TE = 1900/4.38ms, flip angle = 7. T2 images of 64 axial slices were obtained at resolution of 1.25
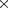
1.25
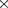
1.95mm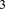, TR/TE = 7380/119ms, flip angle = 150. Preprocessing steps such as skull stripping [19] and bias correction [20] were performed. The skull-stripped results were then reviewed by a trained rater to manually edit, by using ITK-SNAP [21], to ensure the actual removal of non-brain tissues. Diffusion weighted images consisting of 60 axial slices (2 mm in thickness) were scanned with imaging parameters: TR/TE = 7680/82ms, matrix size = 128
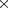
96, 42 non-collinear diffusion gradients, diffusion weighting b = 1000s/mm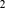. Seven non-diffusion-weighted reference scans were also acquired. The diffusion tensor images were reconstructed and the respective FA images were computed. T2 and FA images were linearly aligned to their T1 images and were resampled with a 1
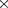
1
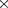
1 mm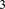 resolution before further processing.

**Figure 6:**
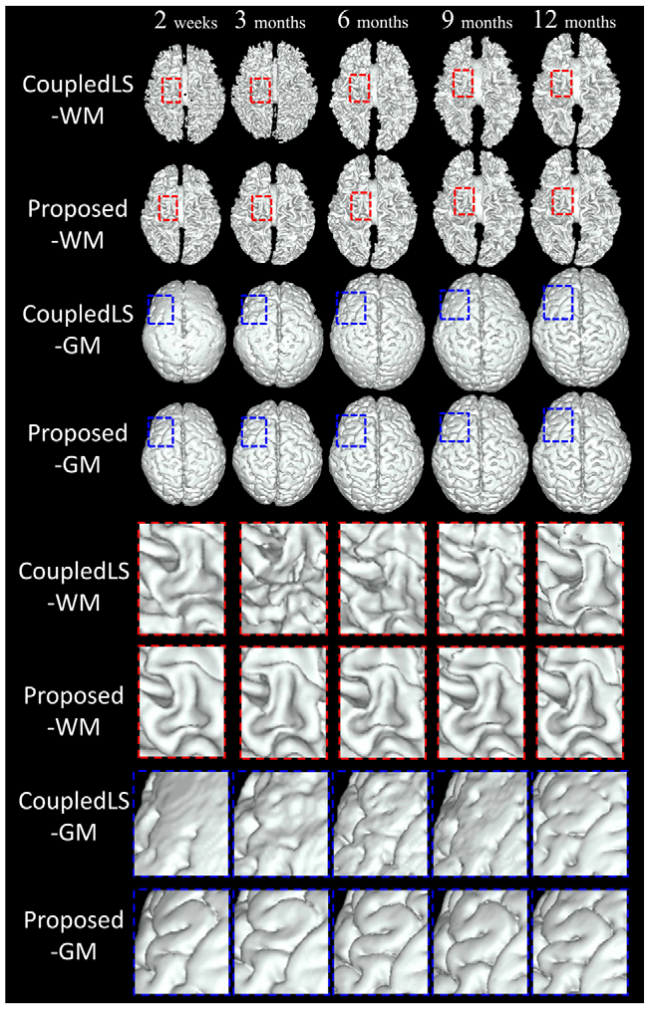
Surface comparison between the coupled level sets (CoupledLS) [**22**] and the proposed method. The lower part shows the zoomed views of the upper part.

### Overview of the Proposed Method

The proposed method utilizes multi-modality information, a cortical thickness constraint, and a longitudinal constraint to derive an accurate and consistent segmentation of serial infant brain MR images. An overview of the proposed framework is shown in Fig. 2. The framework consists of two steps: (1) robust segmentation of each time-point image based on coupled level sets [22]; and (2) iterative 4D registration and segmentation. In the following subsections, we will discuss each component in detail.

**Figure 7:**
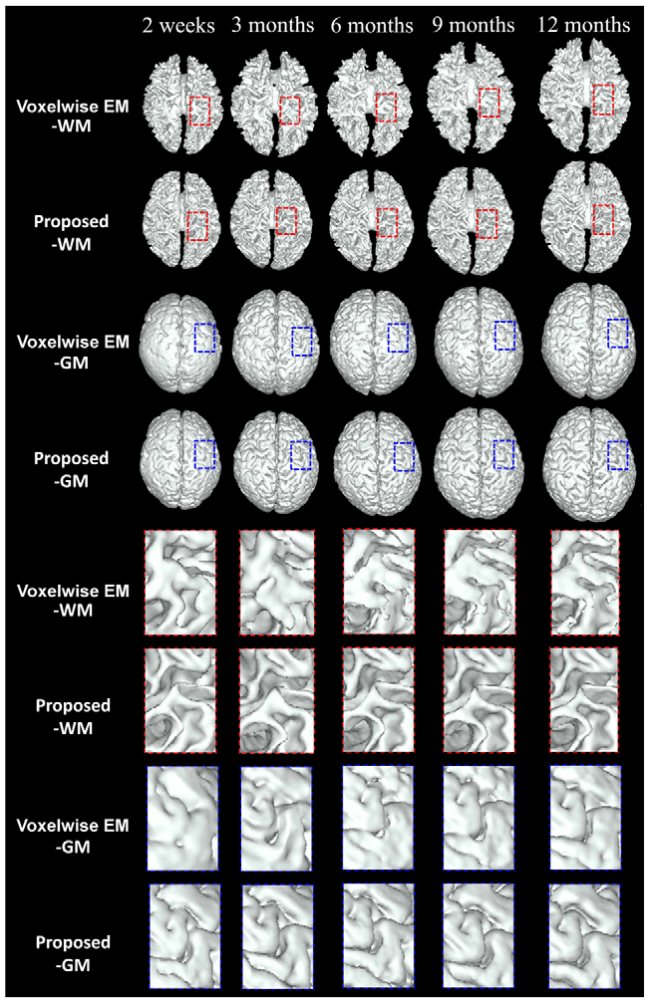
Surface comparison between Voxelwise Expectation-Maximization (EM) method [**12**] **and the proposed method.** The lower part shows the zoomed views of the upper part. From left to right: results at 2 weeks, 3, 6, 9 and 12 months.

**Figure 8:**
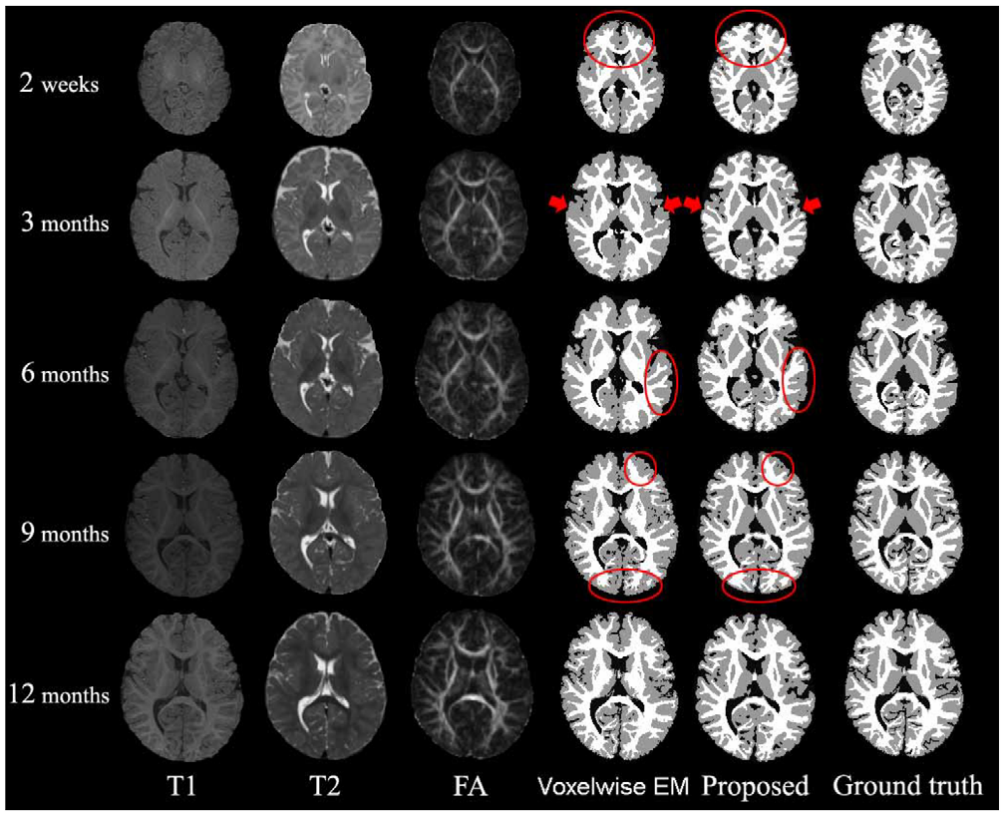
Comparisons of the automated segmentation results with the ground truth. From left to right: original T1, T2, FA images and results of Voxelwise Expectation-Maximization (EM) [12], proposed and manual ground truth. From top to bottom, images acquired at 2 weeks, 3, 6, 9 and 12 months.

**Figure 9:**
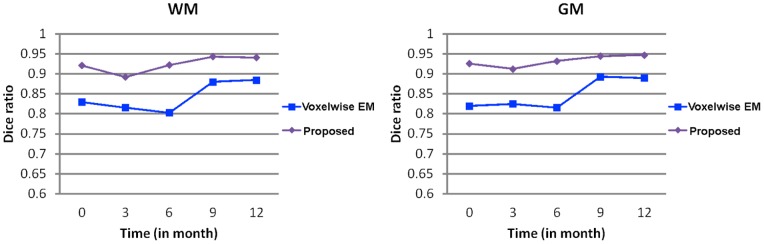
Averaged Dice ratios between manual ground truth and automated segmentations derived by Voxelwise Expectation-Maximization (EM) [**12**] and the proposed method. The proposed method achieves significant (p
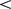
0.01) higher Dice ratio at all time-points.

**Figure 10:**
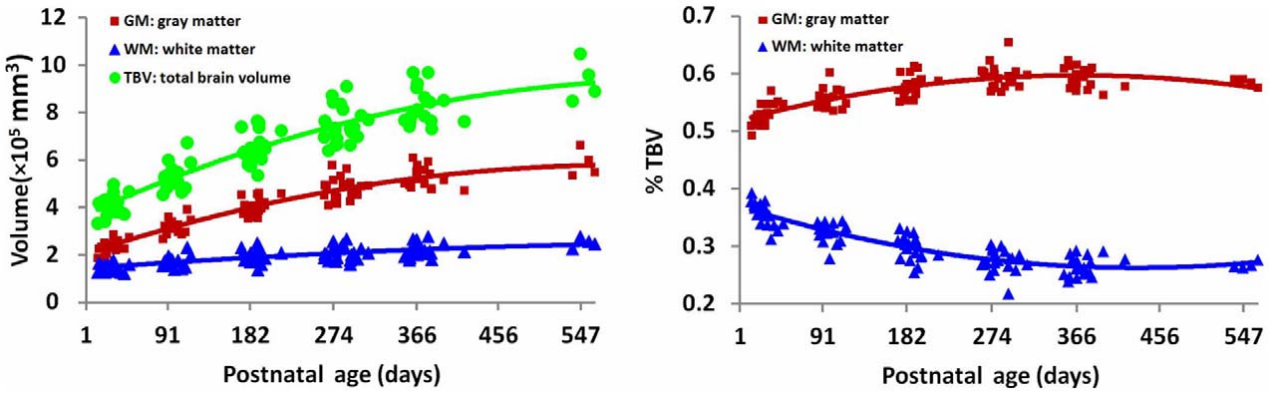
*Left*: Scatterplots of TBV, cortical GM and WM volumes by age at scan. *Right*: Scatterplots of cortical GM and WM volumes in terms of percentage of the total brain volume.

### Multi-modality Data Fitting Term

To robustly segment each time-point image, we make optimal use of T1, T2 and FA images. Let 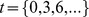 be the age (months) of an infant at scan, and index 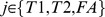 be the modality, i.e., T1, T2 and FA. Let 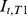, 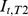, and 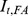 denote the T1, T2 and FA images at time-point 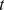. In this paper, the level set function takes negative values outside of the zero-level-set and positive values inside of the zero-level-set. Denoting the level set functions as 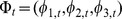 and with the help of the Heaviside function 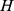, the regions corresponding to WM, GM, CSF and the background, i.e., 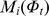, 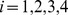, are defined respectively as 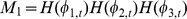, 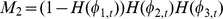, 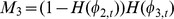, and 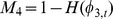, as illustrated in left part of Fig. 3(a). The data fitting energy using both local intensity distribution fitting [22] and population-atlas *prior* 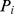 is first defined as,
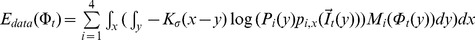
(1)where 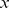 (or 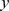) is a voxel in the image domain, 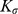 is a Gaussian kernel (with scale 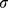) to control the size of the local region [23]–[26], and 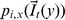 is the probability density of 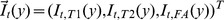 for the tissue class 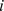. We use the atlas proposed in [10], which was constructed from a unique dataset including 95 normal infant subjects with age from 2 weeks to 2 years old. To take advantage of information given by different imaging modalities (T1, T2 and FA), we represent the distribution of 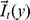 as a multivariate normal (or Gaussian) distribution with mean 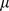 and covariance matrix 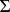,



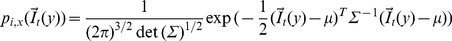
(2)


### Cortical Thickness Constraint Term

As pointed out in [27], [28], the variation of regional cortical thickness is smooth. This observation can be used to constrain surface evolution. To utilize this information, we designed a coupled surface model to constrain the distance of zeros level surfaces of 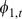 and 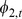 within a predefined range 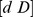, where 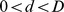. For simplicity of notation, we let 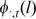 denote the level sets 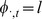. As illustrated in the left part of Fig. 3(a), the zero-level-sets of 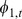 and 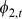, i.e., 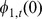 and 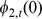, indicate the interfaces of WM-GM and GM-CSF, respectively. As the WM is surrounded by the GM, 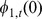 should be interior to 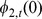 and should fall between the level sets of 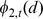 and 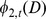 for the thickness to be reasonable. Based on this observation, we define a new cortical thickness constraint term [17] for 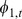,
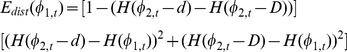
(3)


In a similar way, we can define a cortical thickness constraint term [17] for 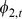,
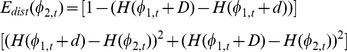
(4)


Therefore, we can define the following energy functional for initial segmentation for each time-point image,
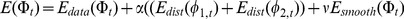
(5)where 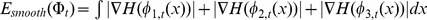 is the length regularization term to maintain a smooth contour/surface during evolution; 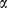 and 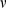 are the blending parameters. The energy (5) can only deal with a single time-point image, and thus cannot benefit from the longitudinal data. In the following, we will propose a longitudinally guided level sets for consistent segmentation.

### 4D Level Set Segmentation

The first year of life is the most dynamic phase of postnatal brain development. However, major sulci and gyri in the adult brain are already present since birth and are retained during early brain development [16], [29]–[31]. Therefore, we can include a longitudinal constraint term or temporal consistency term to better guide the segmentation. For each time-point 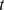, by using 4D registration algorithm, segmentation results from other time points 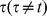 can be registered into the space of the image at the current time-point 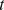. The level set functions 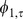 and 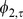 can be similarly warped based on the same deformation fields. Let the warped version of 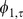 and 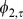 be 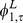 and 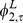, respectively. The distance between the zero-level-surface of 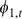 (or 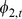) and 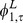 (or 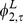) is constrained in a certain range. As illustrated in Fig. 3(a), the evolutions of 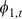 and 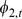 are not only influenced by the information from the current image but is also adaptively constrained by longitudinal information from another time-point 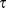 image weighted by 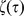. This longitudinal constraint serves to better guide tissue segmentation, and also ensures that the segmented cortical surfaces of the serial infant images of the same subject are consistent with each other.

Similar to Eqs. (3) and (4), we can also constrain the distance between the zero level surfaces of 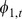 (or 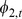) and 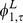 (or 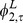). Here, we first consider 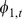. Since the cortex is under rapid development in the first two years and registration across the time-points is not exact, we need to allow the displacement between zero level surfaces of 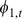 (or 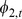) and 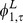 (or 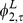). Let the allowed longitudinal range of variation be 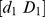 with 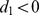 and 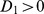, we constrain the zero level sets 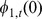 to fall between the level sets 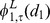 and 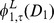. We can define the longitudinal constraint term as,
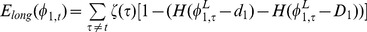
(6)

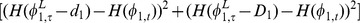
where 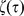 is the weight of each time-point, as shown in Fig. 3(b). Thus, the segmentation of each time-point image will be influenced by its neighboring time-point images. The last time-point image usually has good contrast (see Fig. 1), therefore, its influence will be propagated further and has a greater impact on guiding the segmentation of other time-points. Segmentation of images with lower contrast (e.g., 3, 6, 9 months) can hence be guided by images with higher contrast (e.g., 2 weeks and 12 months).

Similarly, for 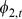, the longitudinal constraint term can be defined as,
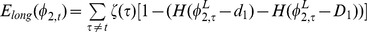


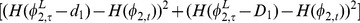
(7)


Finally, we can define the longitudinally guided level-sets energy, which combines local information from T1, T2 and FA images, cortical thickness constraint term, and longitudinal constraint term, as 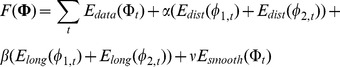
(8)where 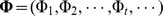, 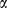, 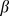 and 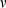 are the blending parameters. It is worth noting that, without FA image, if 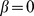, the energy functional will be the same as the coupled level sets (CoupledLS) [22]; if 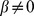 and only two time-points, e.g., year0 and year2, this energy will be the same as the energy proposed in [17]. Therefore, 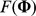 can be considered as a general framework for infant brain MR image segmentation, which actually can deal with single/multiple time-points, and single/multiple image modalities.

To effectively minimize this energy with respect to 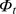, we can rewrite it as,
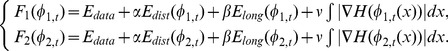
(9)


The energy function 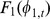 and 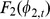 with respect to 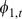, 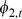 and 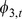 can be easily minimized by using calculus of variations.

The iterative procedure is summarized in Algorithm. 1.

**Algorithm 1** 4D Multi-modality Image Segmentation Using Level Sets

Initial segmentation by applying level-set based segmentations on each time-points (Eq. (5));

**while** convergence criteria is not met **do**

Building 4D correspondences across time-points by using 4D registration [32] based on the

segmentation results;

Longitudinal segmentation: update 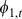, 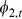 and 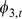 using constraints from neighboring

time-points by minimization of energy (Eq. (8)).

**end while**

## Results

To validate our proposed method, we apply it to a group of 28 infants, each scanned at 5 time points: 2 weeks, 3, 6, 9, and 12 months (or older than 12 months). In our experiments, we set the allowable cortical thickness to 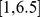
mm, the allowable longitudinal constraint range to 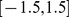
mm, 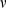 = 0.5, 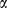 = 0.25, and 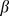 = 0.5. The functions 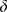 and 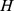 are regularized as in [33]. The level set functions are reinitialized as the signed distance functions at every iteration by using the fast marching method [34]. To measure the overlap rate between the two segmentations 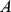 and 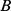, we employ the Dice ratio (DR), defined as 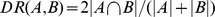. DR ranges from 0 to 1, corresponding to the worst and the best agreement between labels of two segmentations.

### Importance of FA Information

To demonstrate the benefit of incorporating the FA image in the proposed method, we first compare the performance of the CoupledLS [22] for cases when only T1 and T2 images are used and when T1, T2, and FA images are used. These are referred to as *CoupledLS(T1+T2)* and *CoupledLS(T1+T2+FA)*, respectively. Fig. 4 shows the segmentation results for a randomly selected subject with the original images are shown in Fig. 1. The manual segmentations, performed by the trained neuroanatomist, are shown in the last row. The boundaries of neonatal images at the isointense stage are quite fuzzy. The neuroanatomist will use the aligned last time-point (e.g., 1.5-years-old) image as a reference to delineate the boundaries. It can be clearly seen that the *CoupledLS(T1+T2+FA)* (the second row) yields more accurate results than *CoupledLS(T1+T2)* (the first row). For images acquired from the 6-month-olds, the image contrast is quite low. As a result, without information from the FA images, *CoupledLS(T1+T2)* cannot obtain an accurate result (see the blue circled region). Next, we compared the proposed method with the method proposed in [17] *LongLS(T1+T2)* in which T1 and T2 images are used. The results are shown in the third and fourth rows, respectively. It can be clearly seen that the proposed method using T1+T2+FA yields more accurate results, e.g., in the areas marked by the red circles. The mean and standard deviation of DR values of the WM, GM and CSF segmentations of all 28 subjects are shown in Fig. 5. It can be observed that both the CoupledLS and the proposed method achieve much higher accuracy with the help of the FA image.

### Coupled Level Sets (3D) vs the Proposed Method (4D)

To demonstrate the advantages of the proposed method in terms of consistency, in this section, we make comparisons between the CoupledLS [22] working only on a single time-point image and the proposed method working on longitudinal images. For fair comparison, we utilize T1+T1+FA images for both CoupledLS and the proposed method. Since CoulpedLS works on the 3D image individually, the temporal consistency cannot be guaranteed. The 3D rendering of the WM/GM and GM/CSF surfaces are shown in Fig. 6. From the zoomed views (the bottom four rows), it can be seen that the CoupledLS cannot achieve consistent results for serial infant images, while the results of the proposed method are much more consistent. The average DR values on all 28 subjects are also shown in Fig. 5, again demonstrating the advantage of the proposed method.

### Voxelwise EM Method (4D) vs the Proposed Method (4D)

In this section, we make comparison with the method proposed in [12], which was the first method proposed for the segmentation of serial infant brain MR images. This method is a voxelwise Expectation-Maximization (EM) algorithm utilizing subject-specific prior atlases adaptively adjusted by the longitudinal constraints between time-points. Intrinsically, it cannot provide smooth and closed contours/surfaces as final segmentation outcome and cannot guarantee longitudinally consistent segmentation results, although temporal consistency was considered (see Fig. 7). Fig. 8 presents typical segmentation results on a randomly selected subject. Compared with the results of the voxelwise EM, the results of the proposed method are much more consistent with the ground truth, as indicated by the red marks. Fig. 7 presents the surfaces derived by these two methods. It can be clearly seen from the lower zoomed views that the proposed method achieves much more consistent results than the voxelwise EM method. The average DR values are shown in Fig. 9, again demonstrating the advantage of the proposed method in terms of accuracy.

### Volume Analysis

The proposed segmentation method has been validated on 28 subjects. The GM and WM volumes are plotted on the left of Fig. 10. The GM volume increased by 151%, and the WM 55% in the first year of life. The total brain volume (TBV), which was calculated by combining total GM and WM, increased by 115% in the first year of life, while only 11% in the following half year. The volumes, when normalized by TBV, can be used to reflect growth relative to the full brain. As shown in the right of Fig. 10, the percentage of GM increased significantly in the first year, while the WM percentage decreased. Both of them remained relatively constant in the following half year. Our findings are in agreement with a previous study [11].

## Discussion

In this paper, we have presented a longitudinally guided level set method for segmentation of serial infant brain MR images. Combined information from T1, T2 and FA images are utilized by the proposed method. 4D constraint is introduced to ensure consistency across time points. The proposed method has been tested on 28 subjects and high overlap ratio was obtained in comparison to manual segmentations. Extensive comparisons with voxel-based methods and non-4D methods also demonstrate the advantages of the proposed method in terms of accuracy and consistency. The source code and software of the proposed method have been released in NITRC (http://www.nitrc.org/projects/ibeat). Up to now, there have been more than 190 downloads, after it was released in December, 2011.

The rapid growth of the total brain volume in the first year of life reveals that this is a critical period of brain development. This may be a period of developmental vulnerability, but may also be a period of therapeutic interventions with the greatest positive effect. Meanwhile, the relatively faster growing trend of GM compared with WM growth suggests that early growth is dominated primarily by GM. The findings are consistent with a previous study [11].

In this paper, the FA image, which provides better WM contrast [14], has been utilized to guide image segmentation, especially for the 6–8 month old image. The most related work was presented in [17], in which the focus of segmentation was on neonatal (less than 2-month-old) images. That method only utilizes T1-weighted follow-up images for segmentation of T2-weighted neonatal images. The current work extends the previous work [17] in two ways. First, in this paper, we propose a 4D segmentation framework that focuses on the segmentation of the serial infant images, from 2 weeks up to 1.5 years of age. Second, images from multiple modalities (T1, T2 and FA) are utilized in this framework for the accurate segmentation, especially for images of the 6-month-olds, which has lowest brain tissue contrast.

The cortical thicknesses range 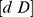 from post-mortem data in adults are in the range of 1.3–4.5 mm [35]–[37]. Although, to the best of our knowledge, there are currently no studies measuring the physical cortical thickness in first year of infant brain, we conservatively set the acceptable range 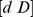 as 1–6.5 mm. For the longitudinal range of variation 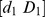, since the cortex is under rapid development in the first two years and registration across the time-points is not exact, we need to allow the displacement between the WM/GM (GM/CSF) boundaries of current time-point and the warped boundary from the other time-points. In this paper, we tested the displacement from 0 mm to 3 mm. We observed an inverted, flat bottomed U-shaped curve for the Dice ratio with a peak at 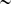
1.5 mm. Therefore, we set the longitudinal range of variation 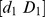 as 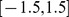 mm. The other weighting parameters 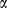, 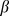 and 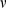 were set based on the similar strategy.

There are many options for 4D registration methods, however, since the main focus of this paper is on segmentation, comparison of the different registration methods is out of scope of the current paper. In this paper, we adopt a 4D HAMMER [32] registration method. In our future work, different registration methods can be evaluated.

## References

[1] Warfield SK, Kaus M, Jolesz FA, Kikinis R (2000) Adaptive, template moderated, spatially varying statistical classification. Med Imag Anal 7: 43–55. 10.1016/s1361-8415(00)00003-7 10972320

[2] Cocosco CA, Zijdenbos AP, Evans AC (2003) A fully automatic and robust brain MRI tissue classification method. Medical Image Analysis 7: 513–527. 14561555 10.1016/s1361-8415(03)00037-9

[3] Prastawa M, Gilmore JH, Lin W, Gerig G (2005) Automatic segmentation of MR images of the developing newborn brain. Med Imag Anal 9: 457–466. 10.1016/j.media.2005.05.007 16019252

[4] Weisenfeld NI, Warfield SK (2009) Automatic segmentation of newborn brain MRI. NeuroImage 47: 564–572. 19409502 10.1016/j.neuroimage.2009.04.068 PMC2945911

[5] Shi F, Fan Y, Tang S, Gilmore JH, Lin W, et al (2010) Neonatal brain image segmentation in longitudinal MRI studies. NeuroImage 49: 391–400. 19660558 10.1016/j.neuroimage.2009.07.066 PMC2764995

[6] Shi F, Shen D, Yap P, Fan Y, Cheng J, et al (2010) Cents: Cortical enhanced neonatal tissue segmentation. Hum Brain Mapp 32: 382–396. 10.1002/hbm.21023 PMC2976792 20690143

[7] Shi F, Yap PT, Fan Y, Gilmore JH, Lin W, et al (2010) Construction of multi-region-multi-reference atlases for neonatal brain mri segmentation. NeuroImage 51: 684–693. 20171290 10.1016/j.neuroimage.2010.02.025 PMC2856707

[8] Xue H, Srinivasan L, Jiang S, Rutherford M, Edwards AD, et al (2007) Automatic segmentation and reconstruction of the cortex from neonatal MRI. NeuroImage 38: 461–477. 17888685 10.1016/j.neuroimage.2007.07.030

[9] Kuklisova-Murgasova M, Aljabar P, Srinivasan L, Counsell SJ, Doria V, et al (2011) A dynamic 4d probabilistic atlas of the developing brain. NeuroImage 54: 2750–2763. 20969966 10.1016/j.neuroimage.2010.10.019

[10] Shi F, Yap PT, Wu G, Jia H, Gilmore JH, et al (2011) Infant brain atlases from neonates to 1- and 2-year-olds. PLoS ONE 6: e18746. 21533194 10.1371/journal.pone.0018746 PMC3077403

[11] Knickmeyer RC, Gouttard S, Kang C, Evans D, Wilber K, et al (2008) A structural MRI study of human brain development from birth to 2 years. J Neurosci 28: 12176–12182. 19020011 10.1523/JNEUROSCI.3479-08.2008 PMC2884385

[12] Shi F, Yap P, Gilmore J, Lin W, Shen D (2010) Spatial-temporal constraint for segmentation of serial infant brain MR images. In: MIAR. 42–50.

[13] Dietrich R, Bradley WG, Zaragoza EJ 4th, Otto RJ, Taira RK, et al (1988) MR evaluation of early myelination patterns in normal and developmentally delayed infants. AJR Am J Roentgenol 150: 889–896. 2450448 10.2214/ajr.150.4.889

[14] Liu T, Li H, Wong K, Tarokh A, Guo L, et al (2007) Brain tissue segmentation based on DTI data. NeuroImage 38: 114–123. 17804258 10.1016/j.neuroimage.2007.07.002 PMC2430665

[15] Osher S, Sethian J (1988) Fronts propagating with curvature-dependent speed: algorithms based on Hamilton-Jacobi formulations. J Comp Phys 79: 12–49.

[16] Armstrong E, Schleicher A, Omran H, Curtis M, Zilles K (1995) The ontogeny of human gyrifica-tion. Cerebral Cortex 5: 56–63. 7719130 10.1093/cercor/5.1.56

[17] Wang L, Shi F, Yap PT, Lin W, Gilmore JH, et al.. (2011) Longitudinally guided level sets for consistent tissue segmentation of neonates. Human Brain Mapping. 10.1002/hbm.21486 PMC4855279 22140029

[18] Gilmore JH, Lin W, Prastawa MW, Looney CB, Vetsa YSK, et al (2007) Regional gray mat-ter growth, sexual dimorphism, and cerebral asymmetry in the neonatal brain. The Journal of Neuroscience 27: 1255–1260. 17287499 10.1523/JNEUROSCI.3339-06.2007 PMC2886661

[19] Shi F, Wang L, Dai Y, Gilmore JH, Lin W, et al.. (2012) Pediatric brain extraction using learning-based meta-algorithm. Neuroimage. 10.1016/j.neuroimage.2012.05.042 PMC3408835 22634859

[20] Sled J, Zijdenbos A, Evans A (1998) A nonparametric method for automatic correction of intensity nonuniformity in MRI data. IEEE TMI 17: 87–97. 10.1109/42.668698 9617910

[21] Yushkevich PA, Piven J, Cody Hazlett H, Gimpel Smith R, Ho S, et al (2006) User-guided 3D active contour segmentation of anatomical structures: Significantly improved efficiency and reliability. Neuroimage 31: 1116–1128. 16545965 10.1016/j.neuroimage.2006.01.015

[22] Wang L, Shi F, Lin W, Gilmore JH, Shen D (2011) Automatic segmentation of neonatal images using convex optimization and coupled level sets. NeuroImage 58: 805–817. 21763443 10.1016/j.neuroimage.2011.06.064 PMC3166374

[23] Li C (2006) Active contours with local binary fitting energy. In: IMA Workshop on New Mathe-matics and Algorithms for 3-D Image Analysis.

[24] Li C, Huang R, Ding Z, Gatenby C, Metaxas D, et al.. (2008) A variational level set approach to segmentation and bias correction of medical images with intensity inhomogeneity. In: MICCAI. volume LNCS 5242, Part II, 1083–1091. 10.1007/978-3-540-85990-1_130 PMC2782702 18982712

[25] Li C, Kao C, Gore J, Ding Z (2007) Implicit active contours driven by local binary fitting energy. In: CVPR. 1–7.

[26] Li C, Kao C, Gore JC, Ding Z (2008) Minimization of region-scalable fitting energy for image segmentation. IEEE Trans Image Process 17: 1940–1949. 18784040 10.1109/TIP.2008.2002304 PMC2720140

[27] Zeng X, Staib L, Schultz R, Duncan J (1999) Segmentation and measurement of the cortex from 3D MR images using coupled surfaces propagation. IEEE Trans Med Imag 18: 100–111. 10.1109/42.811276 10628952

[28] Goldenberg R, Kimmel R, Rivlin E, Rudzsky M (2002) Cortex segmentation: a fast variational geometric approach. IEEE Trans Med Imag 21: 1544–1551. 10.1109/TMI.2002.806594 12588038

[29] Abe S, Takagi K, Yamamoto T, Okuhata Y, Kato T (2003) Assessment of cortical gyrus and sulcus formation using mr images in normal fetuses. Prenatal Diagnosis 23: 225–231. 12627424 10.1002/pd.561

[30] Dubois J, Benders M, Cachia A, Lazeyras F, Ha-vinh R, et al (2008) Mapping the early cortical folding process in the preterm newborn brain. Cerebral Cortex 18: 1444–1454. 17934189 10.1093/cercor/bhm180

[31] Hill J, Dierker D, Neil J, Inder T, Knutsen A, et al (2010) A surface-based analysis of hemi-spheric asymmetries and folding of cerebral cortex in term-born human infants. The Journal of Neuroscience 30: 2268–2276. 20147553 10.1523/JNEUROSCI.4682-09.2010 PMC2836191

[32] Shen D, Davatzikos C (2004) Measuring temporal morphological changes robustly in brain MR images via 4-dimensional template warping. NeuroImage 21: 1508–1517. 15050575 10.1016/j.neuroimage.2003.12.015

[33] Chan T, Vese L (2001) Active contours without edges. IEEE TIP 10: 266–277. 10.1109/83.902291 18249617

[34] Sethian J (1999) Level Set Methods and Fast Marching Methods. Cambridge University Press, Cambridge.

[35] von Economo C (1929) The cytoarchitectonics of the human cerebral cortex. London: Oxford University Press.

[36] Rockel A, Hiorns R, Powel T (1980) The basic uniformity in structure of the neocortex. Brain 103: 221–224. 6772266 10.1093/brain/103.2.221

[37] Henery C, Mayhew T (1989) The cerebrum and cerebellum of the fixed human brain: efficient and unbiased estimates of volumes and cortical surfaces areas. J Anat 167: 167–180. 2630531 PMC1256830

